# The Relationship between Social Capital and Quality of Life among Patients Referring to Diabetes Centers in Isfahan, Iran

**DOI:** 10.1155/2018/9353858

**Published:** 2018-03-07

**Authors:** Mahmoud Keyvanara, Maryam Afshari, Elham Dezfoulian

**Affiliations:** Social determinants of Health Research Center, Isfahan University of Medical Sciences, Isfahan, Iran

## Abstract

**Background:**

Studies have shown that the relationship between social capital (SC) and quality of life (QOL) has an important role in care, prevention, and treatment of some patients. The present study was conducted with the aim to determine the relationship between social capital and QOL of patients with type 2 diabetes.

**Materials and Methods:**

This descriptive, correlational study was conducted on 215 individuals selected through quota sampling. To assess social capital, the Social Capital Questionnaire was used, and to evaluate the QOL of patients with type 2 diabetes, the Diabetes Quality of Life (DQOL) Brief Clinical Inventory was used. Data were analyzed using the Pearson correlation and regression analysis.

**Results:**

A significant relationship was observed between QOL and social capital in patients with diabetes. Moreover, social capital explained 14% of variance in QOL and with the addition of other accompanying diseases, this was increased to 19%.

**Conclusion:**

The results of this study can be useful for health care providers to improve the health of patients with diabetes. They also help patients to better manage and cope with their illness.

## 1. Introduction

The chronic nature of diabetes mellitus (DM) affects patients' body, mind, and individual and social performance and threatens their “quality of life” (QOL). This chronic disease disrupts the patient's family life and outlook on the future [1]. DM threatens individual's independence and gives them a sense of being different from others. This causes psychological stress and impacts the individual's QOL [2]. One of the important goals in the treatment of this disease is the self-management of diabetes. The variable of QOL significantly affects self-care and self-management of disease. Individuals who are satisfied and happy with their life and do not have a feeling of displeasure with being ill and have more energy for self-care. Good self-care results in the individual feeling better and maintaining his/her health, and thus, higher QOL. Hence, this self-reinforcing positive cycle will continue [3]. The importance of QOL in patients with chronic diseases is that some have recognized it as the most important goal of treatment interventions [4]. One of the main theories on the improvement of QOL among patients is related to the social determinants of health, in particular, social capital. Research has shown that social capital is effective on the reduction of high-risk health behaviors. Social capital has been effective on mortality, psychological disorders, and criminal behavior. Its increase results in the improvement of QOL [5]. Previous studies have reported a relationship between social capital and QOL of older people [6], adults [7], patients with fibromyalgia (FM) [8], multiple sclerosis (MS) [9], women [2, 10], and patients with AIDS [11]. A study on social capital and QOL in patients with type 2 diabetes in China showed that although social capital and QOL of these patients were low, the cognitive dimension of social capital was correlated with the QOL of these patients [12]. Another study on the relationship between social capital and glycemic control showed that only the neighborhood connection dimension of social capital was correlated with glycemic control [13]. Other studies have shown that higher social capital may prevent obesity and diabetes [14]. Researchers found that there was not a significant relationship between variables of social capital, social support, income, and age with self-care in diabetic patients [15]. Nevertheless, the variables of gender, time since diagnosis, and education have a significant relationship with self-care in patients with type 2 diabetes. Koetsenruijter et al., in their cross-sectional study in 2015 on 1692 patients with type 2 diabetes from 6 European countries, found that the wide information network, emotional network, and the presence in social organizations and communities are related to self-care capabilities and capacities of these patients [16]. Moreover, a strong relationship exists between self-care capacities of patients with low education levels and information support. Emotional support has a greater impact on the self-care capacities of individuals with high education levels [16]. Henderson et al. performed 28 semistructured interviews with patients in Adelaide in Southern Australia (an area with a low socioeconomic status) [17]. They found that a portion of structural issues and barriers regarding self-care in patients with type 2 diabetes is related to limited access to social, economic, and knowledge resources. Diabetes is a physical illness, but behavioral and social factors also play a role in its onset. Due to the chronic nature of diabetes, it could affect on patient's daily lives. Therefore, it is necessary to take into account the role of social and behavioral factors. Social capital is a valuable resource that enables patient participation in social activities and a sense of cohesion with others. It may provide hope for the future and trust in others in supporting and coping with diabetes.

Based on a report by the Iranian Ministry of Health and Medical Education, diabetes is currently the sixth cause of mortality in the country [18]. More than 4 million individuals in Iran suffer from diabetes, and this figure has tripled every 15 years. The World Health Organization (WHO) has predicted that this rate will reach approximately 7 million individuals by 2030 if no effective measures are taken to prevent and treat diabetes [19]. This disease has short-term and long-term complications as well as direct and indirect costs of the treatment. Therefore, studying factors effective in the management of this disease is of grave importance. Cultural, social, and demographic changes have occurred in recent decades in Iran, including an aging population, changes in nutritional habits, increasing urbanization, and a decrease in physical activity which have been increasing for people with diabetes. It has been claimed that in recent years, social capital has declined in Iran while the number of patients with diabetes has increased. Parts of studies in Iran showed social support [20], and social capital [21] have an undeniable impact on diabetes management. The others also showed that contextual variables are related to the QOL of patients with DM [22]. Although these studies have emphasized social factors, the present study aimed to evaluate the relationship variables of social capital with the QOL of patients with DM.

## 2. Materials and Methods

### 2.1. Subjects and Sampling Methods

The following descriptive-correlational study was conducted in Isfahan, Iran, in 2016. The statistical population consisted of all patients with type 2 diabetes referred to diabetes clinics affiliated with health center numbers 1 and 2. The subjects consisted of 215 individuals selected through quota sampling. After determining the quota of each center, subjects were selected through convenience sampling. The inclusion criteria consisted of a definitive diagnosis of diabetes and lack of chronic diseases such as cancer, asthma, spinal cord injury (SCI), and congestive heart failure.

### 2.2. Measures

The study tools consisted of a demographic checklist, the Social Capital Questionnaire [23], and the Diabetes Quality of Life (DQOL) questionnaire [24]. The Social Capital Questionnaire contains 31 questions which evaluated social capital in the 8 subscales of participation in Isfahan (7 questions), proactivity in a social context (5 questions), feeling of trust and safety (5 questions), neighborhood connection (5 questions), family and friendship connection (3 questions), tolerance of diversity (2 questions), and life values (2 questions). The questions are scored based on the 4-point Likert scale (very low, low, high, and very high). The maximum and minimum total scores of the questionnaire are 124 and 31, respectively; higher scores represent higher social capital. The validity and reliability of this questionnaire were approved through its completion by 1200 individuals in 5 states of Australia by Onyx and Bullen in 2000. In a factor analysis study performed through Varimax method, the correlation coefficient of this questionnaire was reported as 0.25–0.87 and its reliability coefficient as 0.84 [23]. The reliability of this questionnaire was determined among 30 individuals from the statistical population using Cronbach's alpha (*α* = 0.77). The 15-item DQOL was designed by Burroughs et al. in 2004, and its validity and reliability have been assessed (*α* = 0.85). The DQOL consists of the 2 dimensions of the patient's care behavior (8 questions) and satisfaction with disease management (7 questions). The questions are scored based on a 5-point Likert scale ranging from never to always in the care behavior dimension and from completely satisfied to completely dissatisfied in the satisfaction with disease management dimension. The DQOL was translated into Persian by Nasihatkon et al. in 2012. Its validity and reliability using Cronbach's alpha (*α* = 0.63) were defined [25].

### 2.3. Ethical Statement

The required licenses for the performance of the study were obtained from the health centers, and each patient's informed consent was obtained for the completion of the questionnaires. The confidentiality of patients' information was maintained, and their names were not used in the study.

Due to the low literacy level of some patients, each question was read for the patients by the research team and their answers were recorded; thus, a long duration of time was spent on data collect.

### 2.4. Data Analysis

Data were analyzed using the Pearson correlation and multivariate linear regression in SPSS software (version 22, IBM Corporation, Armonk, NY, USA).

## 3. Results

The mean age of the participants was 57.6 ± 10.4 years. In addition, 63 individuals (29.3%) were men and 152 individuals (70.7%) were women. In terms of education level, 136 individuals (63.3%) had an education level lower than high school diploma and 79 individuals (36.7%) had a high school diploma or a university degree. In terms of marital status, 170 individuals (79%) were married, 38 (18%) were widowed, and the rest were single or divorced (Table 1).

The mean QOL score of the participants was 3.4 ± 0.548, and the scores of the dimensions of satisfaction with disease management and care behavior were, respectively, 3.3 ± 0.718 and 3.5 ± 0.728.

Mean total score of social capital was 2.2 ± 0.379. Moreover, the mean scores of the dimensions of participation in local community, proactivity in a social context, feeling of trust and safety, neighborhood connection, family and friendship connection, tolerance of diversity, and life value were, respectively, 1.6 ± 0.508, 2.5 ± 0.514, 2.3 ± 0.502, 2 ± 0.488, 2.4 ± 0.930, 2.4 ± 0.710, and 2.4 ± 0.726 (Table 2).

The results presented in Table 1 show that, based on independent *t*-test results, there was a significant relationship between QOL and gender (*P* = 0.007). In the descriptive statistics, the mean QOL score of men (3.5) compared to that of women (3.3) showed the higher QOL of men. The results of ANOVA showed that the relationship between QOL and age (*P* = 0.106), marital status (*P* = 0.508), occupational status (*P* = 0.950), and education level (*P* = 0.166) was not significant. The results showed a significant relationship between QOL and income (*P* = 0.035).

The results presented in Table 3 illustrate the relationship between social capital and QOL in patients with type 2 diabetes. The Pearson correlation showed a positive and significant relationship between total QOL and total social capital (*P* = 0.001) (correlation coefficient = 0.385). Furthermore, there was a positive and significant relationship between QOL and all dimensions of social capital. The correlation coefficient was, respectively, 4 and 3 in the dimensions of productivity in a social context and tolerance of diversity, which had the highest correlation with the variable of QOL. Moreover, this test showed a positive and significant relationship between total social capital and dimensions of QOL.

The results of correlation test showed that after controlling the variables of gender, age, marital status, and income, there was still a relationship between social capital and QOL (Table 4).

Moreover, the results of multivariate linear regression showed that after entering the variables of social capital, income, gender, marital status, age, education, occupational status, other diseases, and duration of diabetes into the model, the variables of social capital and other diseases remained in the model. Thus, age, marital status, gender, income, education, and occupational status did not impact the relationship between the main variables of the study. Social capital explains 14% of the variance in QOL, and with the addition of other diseases, this rate increased to 19%. This model showed that for each unit of variance in social capital, the QOL score of patients with diabetes, considering the presence of other diseases, increased by 0.362 points.

## 4. Discussion

The results showed that the majority of participants were middle aged or on the threshold of old age (mean age: 57.6 ± 10.4) and the number of women and unemployed individuals was higher than men and employed individuals. Most subjects were married and had an education level of lower than high school diploma. The high number of women and unemployed individuals may be due to the higher rate of referral of women to health centers compared with men and the working hours (usually in the morning) of health centers in Isfahan which reduced the possibility of the presence of employed individuals.

The results also showed a positive and significant relationship between QOL and social capital. This means that an increase in the total social capital scores of patients with type 2 diabetes was accompanied by an increase in their total QOL score. This finding was in accordance with that of the study by Hu et al. [12]. The results of the present study were in agreement with that of previous studies in terms of the relationship between social capital and QOL in different patients, including old age people [6], patients with FM, [8] and MS condition [9]. There were also a relationship between social capital and QOL in patients with AIDS [11], regarding long-term social assistance [26], and the rate of mortality [27]. It is noteworthy that these studies have used different social capital scales and have collected their data in different cultural contexts. The results were not in agreement with studies on the effect of social capital on other variables related to QOL or patients with type 2 diabetes. In a study on the relationship between social capital and glycemic control by Long et al. in Philadelphia, only one of the dimensions of social capital (neighborhood connection) was correlated with glycemic control [13]. In this study, a correlation was observed between total social capital and QOL. However, the correlation of the two dimensions of productivity in a social context (*r* = 0.404) and tolerance of diversity (*r* = 0.283) was higher than other dimensions. The findings of this study were in accordance with that of the study by Farajzadegan et al. in Iran [21] which studied the relationship between social capital and diabetes management.

Holtgrave and Crosby, in their study on American adults with type 2 diabetes, found that social capital was the strongest predictor of obesity and diabetes [14]. The results of this study were in agreement with the present study in terms of the effect of social capital on health-related variables in patients with type 2 diabetes. Nevertheless, the results of the current study were not in agreement with that of the study by Weaver et al. on 97 patients with type 2 diabetes in Canada [15]. They found that social capital, social support, income, and age did not have a significant relationship with self-care in these patients, while gender, time since diagnosis, and education had a significant relationship with self-care.

The results of this study approved the effect of social support on QOL and showed no relationship between time of diagnosis and QOL. Nevertheless, the presence of other accompanying diseases was found to be effective on QOL. The findings of this study are in agreement with that of the study by Koetsenruijter et al. in 6 European countries in terms of a correlation between QOL and education in patients with diabetes [16].

Social capital, through social ties and interpersonal interactions, provides the levels of access for individuals and patients to economic, social, and other resources. This allows patients to have a better life in the community. People who have better social capital are more likely to preserve the material and spiritual properties. The positive effects of social capital will be further when interrelated to the quality of life, greater synergies for improving the lives of patients with chronic diseases—in this study, diabetes condition. The quality of life helps to increase the sensitivity of patients to their own health and seek ways to become aware of the illness, treatment, and control of complications and prevent the onset of the disease. Thus, social capital by improving the quality of life can provide better life for patients with chronic conditions; patients have to live with the disease for many years. In this state, patients are in a situation where their feelings about illness is understood, it is possible to exchange and interact with other which reduce their pressures and concerns, and further hope will lead them to improve morale, lower tension, and increase life expectancy. Overall, patients who achieve better quality of life are considered as capital.

A limitation of the present study was that the study sample was a clinical sample from health centers in the city of Isfahan. Therefore, it did not represent the population of patients with type 2 diabetes of Isfahan. Due to the nature of correlational studies, the relationship between variables was not a definitive relationship and the results of the present study can be completed by further studies. Other variables which may have affected the results of the study (such as lifestyle) were not taken into consideration.

## 5. Conclusion

It can be concluded that the QOL of patients with type 2 diabetes was medium and their social capital was low. In addition, the results of this study approved the positive and significant relationship between social capital and QOL. They also showed that social capital, particularly in the two dimensions of “productivity in a social context” and “tolerance of diversity,” has a positive effect on the QOL of patients. Thus, strategies to strengthen social capital among patients can be effective on their QOL and, thus, on their overall health. Creating a framework for the growth of group activity will facilitate productivity in a social context. In addition, the expansion of the individual relationship network and communication with individuals from different cultural can increase tolerance of diversity. In this sense, emotional and social support increased, and in turn, decreased physical and mental tensions.

## Figures and Tables

**Table 1:** The characteristics of diabetic patients.

Variables		*n* (%)	Mean^∗^ (QOL)	Std. Dev	Sample range	*P* value
Sex	Male	63 (29.3)	3.5	0.60		0.007
	Female	152 (70.7)	3.3	0.52		
Age			57.6	10.4		0.106
Income			2.5	1	1–5	0.035
Educational level^∗∗^	Low	136 (63.3)	3.4	0.501		0.166
	High	79 (36.7)	3.5	0.617		
Marital status	Married	170 (79)				0.508
	Single	45 (21)				
Employment status	Employed	18 (8)				0.950
	Unemployed	197 (92)				
Illness except diabetes		155 (72.1)				<0.001

^∗^Mean values based on summated and average scores. ^∗∗^Having low education = high school and lower. Having high education = high school diploma and upper (university degree).

**Table 2:** Summary statistics on quality of life and social capital.

Variables	Mean	Std. Dev
Quality of life	3.4	0.548
Satisfaction with treatments	3.3	0.718
Self-care behavior	3.5	0.728
Social capital	2.2	0.379
Participation in local community	1.6	0.508
Productivity in a social context	2.5	0.514
Feeling of trust and safety	2.3	0.502
Neighborhood connection	2	0.488
Family and friendship connection	2.4	0.930
Tolerance of diversity	2.4	0.710
Value of life	2.4	0.726

**Table 3:** The correlation between social capital and dimensions of quality of life of patients with type 2 diabetes.

	Quality of life	Social capital	Participation in local community	Productivity in a social context	Feeling of trust and safety	Neighborhood connection	Family and friendship connection	Tolerance of diversity	Life value	Self-care behavior	Satisfaction with treatments
Quality of life	1										
Social capital	0.385 <0.001	1									
Participation in local community	0.178 <0.001	0.733 <0.001	1								
Productivity in a social context	0.404 <0.001	0.728 <0.001	0.403 <0.001	1							
Feeling of trust and safety	0.266 <0.001	0.729 <0.001	0.403 <0.001	0.488 <0.001	1						
Neighborhood connection	0.256 <0.001	0.747 <0.001	0.526 <0.001	0.441 <0.001	0.440 <0.001	1					
Family and friendship connection	0.218 <0.001	0.617 <0.001	0.302 <0.001	0.312 <0.001	0.321 <0.001	0.385 <0.001	1				
Tolerance of diversity	0.283 <0.001	0.475 <0.001	0.173 0.011	0.314 <0.001	0.310 <0.001	0.353 <0.001	0.221 <0.001	1			
Life value	0.234 <0.001	0.556 <0.001	0.342 <0.001	0.417 <0.001	0.436 <0.001	0.271 <0.001	0.254 <0.001	0.141 0.039	1		
Self-care behavior	0.718 <0.001	0.328 <0.001	0.279 <0.001	0.324 <0.001	0.234 <0.001	0.142 0.038	0.158 0.020	0.136 0.047	0.266 <0.001	1	
Satisfaction with treatments	0.794 <0.001	0.260 <0.001	0.007 .916	0.288 <0.001	0.170 0.013	0.239 <0.001	0.173 0.011	0.288 <0.001	0.099 0.149	0.148 0.030	1

**Table 4:** Regression analysis coefficients for factors effective on quality of life.

Independent variable	*b*	Beta	*R* square	*P* value
Constant value	2.47	—	—	<0.001
Social capital	0.528	0.362	0.148	<0.001
Other diseases	−0.279	−0.227	0.199	<0.001
